# Social media: A new tool for outbreak surveillance

**DOI:** 10.1017/ash.2021.225

**Published:** 2021-11-17

**Authors:** Averi E. Wilson, Christoph U. Lehmann, Sameh N. Saleh, John Hanna, Richard J. Medford

**Affiliations:** 1 Department of Internal Medicine, University of Texas Southwestern Medical Center, Dallas, Texas; 2 Department of Pediatrics, University of Texas Southwestern Medical Center, Dallas, Texas; 3 Clinical Informatics Center, University of Texas Southwestern Medical Center, Dallas, Texas; 4 Division of Infectious Diseases & Geographic Medicine, University of Texas Southwestern Medical Center, Dallas, Texas

## Abstract

Social media platforms allow users to share news, ideas, thoughts, and opinions on a global scale. Data processing methods allow researchers to automate the collection and interpretation of social media posts for efficient and valuable disease surveillance. Data derived from social media and internet search trends have been used successfully for monitoring and forecasting disease outbreaks such as Zika, Dengue, MERS, and Ebola viruses. More recently, data derived from social media have been used to monitor and model disease incidence during the coronavirus disease 2019 (COVID-19) pandemic. We discuss the use of social media for disease surveillance.

Engaging in social media is a popular activity for billions of individuals globally.^1^ A by-product of social media activity is the generation of large quantities of data. Facebook (Menlo Park, CA), for example, produces 4 million posts every minute.^2^ Data processing methods allow researchers to automate the collection and interpretation of social media activity and create valuable knowledge. Social media interaction data have been shown to complement and enhance disease surveillance for infectious diseases such as Zika, Dengue, Middle East Respiratory Syndrome (MERS), and Ebola viruses.^3–7^ During the coronavirus disease 2019 (COVID-19) pandemic, researchers surveilled social media to understand disease activity and public perceptions, resulting in improved modeling of COVID-19 infection rates.^8–15^ Here we describe the history, methodology, and advantages of using social media for surveillance of infectious disease outbreaks.

## Background

### History of social media

Social media is described as an interactive, digital technology that enables creation, sharing, and exchange of information, ideas, opinions, thoughts, and other forms of expression in form of text, emojis, images, audio, and videos via virtual communities and networks. The first social networking and video sharing platform, Bolt, emerged in 1996.^16^ In 2021, 3.96 billion people are active social media users. Globally, the average person has 8.8 social media accounts and spends >2 hours daily engaging in social media. Facebook has 2.7 billion monthly active users and the video platform YouTube has 2 billion active users. With this expansion of social media across the globe, there is growing interest in using social media activity data to gain insight into infectious diseases and pandemics.

### Twitter

Twitter (San Francisco, CA) is a social media platform with 350 million active monthly users^16^ and 187 million daily users. Users post short messages limited to 280 characters, termed “Tweets” that also capture the date, time, and geographic location of the post, the number of users interacting with the post and the types of interaction, and other metadata associated with a tweet and a specific user.^17^ Twitter data are relatively easy for researchers to access in comparison to other platforms. Researchers with a Twitter developer account have access to the Twitter application programming interface (Twitter API), which provides a consistent, programmatic way to retrieve tweets and their metadata.^18^ Available code libraries in various programming languages (eg, tweepy in Python or rtweet in R) enable users to obtain and analyze data obtained from the Twitter API. These tools give researchers and public health entities the potential to analyze and monitor tweets for disease surveillance.

## Natural language processing methods: A brief overview

Natural language processing combines linguistics, artificial intelligence, and machine learning to enable machines to process human language, such as that used in social media posts, and to extract meaning from it.^19^ Social media analysis requires that text is preprocessed prior to analysis. For example, text is “cleaned” by removing hyperlinks, user tags, or words with little analytical value (eg, “is” or “and”). Words are often then lemmatized, or brought back to their root form (ie, “distancing” to “distance” or “viruses” to “virus“), and text is segmented into 1- and 2-word phrases called named unigrams and bigrams, respectively. Words and phrases with extremely low or high frequency can be removed for simplification and dimensionality reduction. Machine-learning algorithms are used to identify clusters of texts grouped by similar words in a process known as topic modeling, which allows researchers to visualize the distribution and frequency of topics over time or to explore more granular analyses for a particular topic cluster.^8^ This methodology requires a level of technical skill; however, companies such as Symplur (Pasadena, CA), an online analytics platform, offer researchers automated natural language processing services to identify and analyze trending language on social media.^20^

## Early use of web-based data

### Infodemiology

Prior to the use of social media for disease surveillance, researchers analyzed Internet search-engine terms to predict disease trends. The concept of “infodemiology” was introduced in 2004 when researchers evaluated the automated analysis of Internet search trends to predict trends in the 2004–2005 Canadian influenza season.^21^ A Google advertisement campaign was created that targeted Canadian Internet users who searched the key words “flu” or “flu symptoms.” An advertisement labeled “Do you have the flu? Fever, Chest discomfort, Weakness, Aches, Headache, Cough?” appeared for these users and contained a link to a patient education website. Historically, Google declined to provide search data to researchers; however, the advertisement allowed researchers to capture measures such as number of advertisement views and number of click-throughs received. Researchers correlated the daily views and clicks with weekly national influenza reports and found that the daily number of clicks correlated with the number of confirmed cases.^21^

### Google Flu Trends

In 2008, Google (Mountainview, CA) developed its own surveillance technology, Google Flu Trends, which tracked search trends to make predictions about influenza activity.^22^ Using Internet protocol addresses to localize searches, Google computed a state-level time series for 50 million common search queries. They used a linear model to compute the log odds of an influenza-like-illness (ILI) as reported by the Centers for Disease Control and Prevention (CDC) and the log odds of an influenza-related search query. Each search query was tested for matching the CDC-reported ILI data for that period. The 45 queries that best matched the ILI data were aggregated to compute a linear prediction model.^22^ Google Flu Trends was initially reported to be 97% accurate with CDC data, but it eventually stopped publishing estimates in 2015 after overestimating flu incidence and office visits between 2011 and 2013.^22^ These overestimations were explained with the prominence of flu in the news (ie, bringing it to people’s attention and into their searches) and the inability to distinguish searches for illnesses with similar symptoms.

## Examples of surveillance using social media

Social media posts reflect what occupies the minds of people worldwide in near real time creating an increase in interconnectivity termed the “global village.”^23^ Social media may be useful for understanding disease outbreaks through a method known as syndromic surveillance.^24^ The CDC defines syndromic surveillance as a method for using health-related data (like symptom groups) to predict the probability of an impending outbreak.^25^ Social media can complement traditional surveillance methods by offering additional data types. For example, the frequency of pertinent hashtags may correlate with disease incidence; geotagged social media entries may indicate crowding and risk of spread; and social media posts can report on school or store closings or supply shortages.^26^ Social media reports on public perceptions of nonpharmacological interventions like “work from home” or “wear a mask” orders may be compared with new infection rates to determine effectiveness and to provide early insight into transmission modalities. During the 2009 H1N1 pandemic, Signorini et al^27^ collected tweets with relevant terms to track disease prevalence and public sentiment regarding the infection and prevention efforts. Social media has also been effective in forecasting (ie, the prediction of an event based on past or present data) and monitoring public perceptions and behaviors during the Zika, Dengue, MERS, and Ebola outbreaks.^3–7^

### Zika virus

From 2015 to 2016, McGough et al attempted to forecast Zika cases in Latin America using Zika-related Google search data, Twitter posts, and the HealthMap digital surveillance systems, which use “online informal sources for disease outbreak monitoring and real-time surveillance of emerging public health threats.”^3,28^ Weekly, these researchers evaluated the percentage of Zika-related Google search terms as well as Zika-related tweets and the reported incidence of Zika on HealthMap. They calculated correlations between these potentially predicting factors and the reported Zika case count for the corresponding week as well as 1–3 weeks in the future to account for possible lag time between online predictors and case reports. Models combining Google search data and Twitter posts with past values had the best predictive accuracy for case reports 1 week in the future.^33^ The use of the autoregressive information (past data) improved prediction accuracy 1 week in the future because current disease incidence and number of infected mosquito vectors have a major impact on future incidence. Models without autoregressive information performed best 2–3 weeks in the future, suggesting that predictions further into the future are less dependent upon current cases and benefit from focusing on Google and Twitter alone. Overall, exclusive Google search models had the lowest error and the addition of Twitter data, where available, improved prediction accuracy.^33^ The addition of HealthMap data did not improve the prediction accuracy, possibly due to the lag time between actual disease cases and their reports on HealthMap.^33^

### Dengue virus

Marques-Toledo et al^4^ used tweets for an early detection and monitoring model of Dengue in Brazil. Tweets with Dengue key words were collected, and a machine-learning algorithm identified tweets suggestive of personal experience with Dengue to exclude parody, opinion, informational, and marketing tweets. Researchers collected the quantity of tweets, date and time of posting, and geographic location from where tweets were posted. Researchers also collected Google search trends for relevant terms and studied the access frequency of Dengue-related Wikipedia articles. Tweets, Google trends, and Wikipedia access showed a strong association with nationally reported Dengue cases.^4^ Tweets better estimated Dengue cases in the present because users were more likely to tweet about illness while symptomatic. Although forecasting was possible, inaccuracies grew as predictions were further into the future. Regions with the highest incidence of Dengue also had more Dengue-related Tweets, indicating that tweets are useful for understanding the geographic distribution of an outbreak. Tweets successfully predicted Dengue incidence at the city level; however, goodness of fit of the tweet model was influenced by factors including disease incidence, local computer and Internet access, income, and education level.^4^ These researchers noted that utilization of social media is a cost-effective way to improve traditional methods of Dengue surveillance, which have classically suffered from underreporting and reporting delays.^4^

### Middle East respiratory syndrome coronavirus

In 2015, during the Middle East respiratory syndrome coronavirus (MERS-CoV) in eastern Asia, researchers evaluated the use of web-based searches and social media activity to monitor MERS-CoV activity.^5^ Researchers correlated the daily frequency of key terms such as “MERS,” “MERS symptoms,” and “MERS hospital” in Google searches and Twitter posts, with daily confirmed and quarantined MERS cases. Peaks in confirmed and quarantined cases occurred a respective 5 and 15 days after peaks in social media and search activity. Social media and search activity were correlated with lag correlations of confirmed cases 0–4 days prior to confirmation.^5^

### Ebola virus

Social media platform data also provided useful insight during the 2014 Ebola outbreak. Researchers used the web-based tool Topsy (a now defunct service that collected and stored all tweets) and Google Search Trends to evaluate trends related to Ebola in social media use.^6^ They found that frequency of the word “Ebola” in tweets and in Google searches worldwide peaked within 24 hours of news events such as major Ebola exposures and reports of Ebola in countries not previously affected.^6^ “Top influencers” were popular news outlets and, although most tweets originated in the United States, African countries dramatically affected by Ebola also had a large social media presence.^6^ Odlum et al^7^ also capitalized on social media–based data to monitor information spread, epidemic detection, and public knowledge and attitudes. They used time series analysis and geographic visualization to understand disease and information dissemination during the outbreak. Tweet volume increased 3–7 days after large news breaks, even in countries with lower adoption of social media. They used natural language processing to analyze tweets mentioning Ebola to examine public attitudes surrounding Ebola and noted fearful tweets increased soon after major news reports.^7^ Their data indicate that social media can be a useful adjunct for early detection of disease, dissemination of information, and understanding of public perception.

## Applications for COVID-19 surveillance

Our review of social media in surveillance of emerging infections shows, less newsworthy illnesses seem better suited for prediction using social media because there is less influence through media reports. A feared disease, such as Ebola, is likely to receive more press reports; thus, its actual effects on the population may be distorted. Despite this media distortion, social media-derived data have provided valuable insights. Tsao et al^10^ reviewed 91 studies regarding social media in the COVID-19 era and modified a novel framework called “Social Media and Public Health Epidemic and Response (SPHERE).”^10,29^

Three main categories describe the use of social media as it relates to COVID-19: (1) “Social media as a contagion and a vector” refers to the “infodemic” or rapid dissemination of pandemic information via social media. (2) “Social media as disease control” refers to efforts by public health organizations to create and disseminate materials educating the public with accurate information about the pandemic via social media. (3) “Social media as surveillance” refers to the monitoring of public attitudes, mental health, and case detection during the pandemic.^10^

Of 81 studies in the third category, only 6 investigated COVID-19 surveillance using social media platforms. Qin et al^11^ collected social media search frequency from a popular Chinese social network (Baidu) for infectious disease key words (eg, dry cough, fever, chest distress, pneumonia, and coronavirus) and used a lagged series of search frequencies to predict suspected case numbers. After evaluating several prediction methods, these researchers used the “subset selection method,” in which 10 of the 50 available predictors that most accurately predicted case numbers were selected and used for analysis. With this method, the number of suspected COVID-19 cases correlated best with search frequencies recorded 6–9 days earlier. Social media search frequencies 10 days earlier correlated best with the number of confirmed COVID-19 cases.^11^ The correlation between social media search indices and suspected COVID-19 cases was stronger than its correlation with PCR confirmed COVID-19 cases.

Li et al^12^ evaluated the prediction strength of search data from Baidu, Google, and Sina Weibo (Chinese social media platform) search engines. They correlated the frequency of 2 search terms, “coronavirus” and “pneumonia,” with daily numbers of suspected and PCR-confirmed COVID-19 cases in China. A correlation was detected with Internet search volume 5–7 days prior to suspected cases and 8–10 days prior to PCR-confirmed cases.^12^ Peng et al^13^ used geotagged data from Sina Weibo to analyze the spatiotemporal distribution of COVID-19 cases in Wuhan, China. Sina Weibo developed a COVID-19 informational channel early in the pandemic to help patients without access to timely treatment. Help seekers provided information such as name, age, address, contact number, and illness details to an Internet form. Spatial distribution of help seekers reflected the severity of infections clustered in families as well as concentrated regional patterns across the city. Regions with higher population density, especially regions with higher density of elderly individuals, had the highest concentration of COVID-19 help seekers.^13^

Machine learning and natural language processing can be used to understand the general public’s perceptions during the pandemic (Public Attitudes – Modified SPHERE framework), and these data can complement surveillance and forecasting efforts. Medford et al^8^ studied Twitter data to understand the pandemic’s effect on the emotions, beliefs, and thoughts of the public.^8^ They extracted relevant Tweets from the weeks prior and after the activation of the CDC Emergency Operations Center using pertinent hashtags such as #2019nCoV, #coronavirus, #nCoV2019, #wuhancaronavirus, and #wuhanvirus. Variables of interest included tweet text, time, included images or links, the type of tweet (retweet or reply), and the number of likes, retweets, and replies. They applied natural language processing methods to prepare tweets for sentiment analysis and topic modeling. Analyses showed tweets most commonly expressed sentiments of fear and surprise. The economic impact of the pandemic was the most discussed topic.^8^ Saleh et al^14^ performed a similar analysis with the goal of understanding public perception of social distancing and found most tweets expressed positive polarity.^14^ Joy was the predominant emotion and was expressed in >50% of tweets. The most common tweet topics were “public opinion and values,” “media and entertainment,” and “quarantine measures and effects.”^14^ Sentiment analysis complements transmission models, as these analyses give researchers insight on public perception on prevention and control measures. Medford et al^8^ also highlighted the utility of this technology for combating misinformation on social media and the associated fear and mistrust. Individuals or autonomous “bots” on social media platforms spread malicious and dangerous misinformation.^30,31^ Natural language processing methods give platforms the ability to recognize potentially misleading posts and counter them with educational information, making them likely a worthwhile investment for organizations such as the World Health Organization (WHO) or the CDC.^8^

The works of Medford et al and Saleh et al are important in the context of Kermack and McKendrick’s “Susceptible, Infected, Removed or Recovered (SIR)” model, which is a well-tested disease transmission model.^32^ The model partitions the population into compartments based on who in the population is “susceptible” to a disease, who is “infected,” and who is “removed” from the population due to past infection resulting in either death or immunity. The model allows researchers to simulate epidemics by using differential equations to model the flow of individuals among the 3 compartments. The model’s behavior is dependent on the inherent infectivity of the disease and the population density of the susceptible population.^33^ This model has been well tested for various scenarios, but it assumes stochastic interaction that is not realistic in populations with a more complex social structure. For example, an infected individual with a large social network may infect more individuals than someone with a small number of contacts. In the context of COVID-19 modeling, assumptions about the proportion of people following social distancing guidelines or participating in vaccination efforts are important for determining the number of individuals susceptible. Natural language processing for analyzing social media posts can improve these assumptions based on the proportion of users posting in favor of practices such as social distancing and vaccination.

Another potential source of disease surveillance derives from crowdfunding sites.^15^ As the number of COVID-19 cases and its economic impact increased in the United States, reports noted an increase in web-based crowdfunding related to its costs. Saleh et al^15^ examined the web-based crowdfunding response in the early stages of the COVID-19 pandemic in the United States using campaigns with narratives on GoFundMe.^15^ A substantial increase in overall crowdfunding campaigns in March was largely attributable to COVID-19–related campaigns. However, as the COVID-19 pandemic progressed, the number of campaigns per COVID-19 case declined more than 10-fold, and there was a lack of a case-dependent response. Saleh et al^15^ concluded web-based crowdfunding appears to be a stopgap for only a minority of campaigners. However, crowdfunding activity may serve an early signal for emerging needs and could aid the provision governmental disaster relief.

## Limitations of social media

Clinical surveillance programs for emerging infectious diseases are labor intensive, costly, and time-consuming, and they require a substantial workforce.^3–7^ Leveraging high volume, publicly available data from social media posts provide a time and cost-efficient alternative to traditional measures. Thus, utilization of social media posts for pandemic surveillance is of increasing relevance as social media use becomes increasingly ubiquitous; however, several limitations must be considered.

Underdeveloped areas, both locally and abroad, with less Internet and technology access, are also areas at high risk of being disproportionately affected by pandemics due to crowding, multigenerational households, and decreased access to information.^34^ Lack of access likely causes underrepresentation of these populations in disease models, further aggravating health disparities already faced. Additionally, natural language processing methods are language dependent and, although researchers can complete their analyses in multiple languages, it would be challenging and expensive to adequately include posts in less common languages or dialects, resulting in further selection bias.

Social media surveillance measures are dependent on natural language processing models, which also present some inherent limitations. Not every social media post with certain language or key words is reflective of an infected individual but may be reflective of interest in the disease or prevalence of the disease in the media. Although the natural language processing model can be optimized to extract the most relevant data, the model may not be able to consistently distinguish between infected and interested users or between serious and sarcastic posts. Post volume sufficient to generate enough signal is important to compensate for noise. Thus, this type of surveillance more difficult to execute in populations with less access to social media.

In conclusion, social media posts have the potential to provide real-time information about disease incidence in the absence of timely testing or disease reporting. Public health entities should consider leveraging these types of data for disease surveillance going forward. Currently, documented studies describe the use of social media for disease surveillance are retrospective in nature and have been carried out in response to an emerging pandemic or epidemic.^3–15^ A major benefit of using social media posts for disease surveillance is the potential for early warning systems. Future work in the field should focus on proactive approaches for monitoring known and emerging infectious diseases. Although studies of surveillance claim that it is cost-effective in comparison to traditional surveillance methods, further research is necessary to determine the comparative cost of employing individuals properly trained in data science and natural language processing.^4^ Additionally, there is untapped potential in the analysis of posts on video-based social media platforms, and these methods should be explored further. Social media data are valuable to surveillance of infectious diseases and will remain a valuable resource for healthcare knowledge generation in the future.
